# *In vivo* characterization of doxycycline-mediated protection of aortic function and structure in a mouse model of Marfan syndrome-associated aortic aneurysm

**DOI:** 10.1038/s41598-018-38235-6

**Published:** 2019-02-14

**Authors:** Jason Z. Cui, Ling Lee, Xiaoye Sheng, Fanny Chu, Christine P. Gibson, Taline Aydinian, David C. Walker, George G. S. Sandor, Pascal Bernatchez, Glen F. Tibbits, Cornelis van Breemen, Mitra Esfandiarei

**Affiliations:** 1 Department of Anesthesiology, Pharmacology and Therapeutics, British Columbia Children’s Hospital Research Institute, University of British Columbia, Vancouver, BC Canada; 2 Department of Biomedical Physiology and Kinesiology, Simon Fraser University, Burnaby, BC Canada; 3 Department of Anesthesiology, Pharmacology and Therapeutics, Centre for Heart Lung Innovation, St. Paul’s Hospital, University of British Columbia, Vancouver, BC Canada; 4 Department of Biomedical Sciences, College of Graduate Studies, Midwestern University, Glendale, Arizona USA; 5 Children’s Heart Centre, British Columbia Children’s Hospital, University of British Columbia, Vancouver, BC Canada; 6 Department of Cardiothoracic Surgery, School of Medicine, Stanford University, Palo Alto, California USA

## Abstract

Aortic aneurysm is the most life-threatening complication in Marfan syndrome (MFS) patients. Doxycycline, a nonselective matrix metalloproteinases inhibitor, was reported to improve the contractile function and elastic fiber structure and organization in a Marfan mouse aorta using *ex vivo* small chamber myography. In this study, we assessed the hypothesis that a long-term treatment with doxycycline would reduce aortic root growth, improve aortic wall elasticity as measured by pulse wave velocity, and improve the ultrastructure of elastic fiber in the mouse model of MFS. In our study, longitudinal measurements of aortic root diameters using high-resolution ultrasound imaging display significantly decreased aortic root diameters and lower pulse wave velocity in doxycycline-treated Marfan mice starting at 6 months as compared to their non-treated MFS counterparts. In addition, at the ultrastructural level, our data show that long-term doxycycline treatment corrects the irregularities of elastic fibers within the aortic wall of Marfan mice to the levels similar to those observed in control subjects. Our findings underscore the key role of matrix metalloproteinases during the progression of aortic aneurysm, and provide new insights into the potential therapeutic value of doxycycline in blocking MFS-associated aortic aneurysm.

## Introduction

Marfan syndrome (MFS) is an autosomal dominant disorder of connective tissue characterized by defects in the cardiovascular, pulmonary, skeletal, and ocular systems, with a frequency of approximately 1 in 3,000–5,000; caused by mutations in a gene that codes for fibrillin-1 (*FBN1*)^1–3^. FBN1 is a large cysteine-rich glycoprotein and is considered as the main structural component of the FBN1-rich microfibrils within the extracellular matrix (ECM), providing a scaffold for elastic fiber formation and maturation^4^. In MFS patients, various degrees of genetic deficiency in FBN1 protein lead to disorganization and destruction of elastic fibers and collagen, causing significant reduction in the load-bearing capacity of the aorta, leading to aortic root dilatation, micro-dissection and rupture^3^.

Previously, using the mouse model of MFS, we were able to show that progression of aortic aneurysm was associated with significant increases in the expression levels of matrix metalloproteinase (MMP)-2 and MMP-9^5–8^. We also reported that MMPs worked as endopeptidases that degraded the majority of ECM components and microfibrils, leading to degradation of elastin fibers, reduction in smooth muscle contractility, and evident signs of endothelial dysfunction^5–8^. Using conventional histology and *ex vivo* small chamber myography technique, we have previously reported that long-term treatment with doxycycline, a nonspecific and general MMPs inhibitor, significantly improves aortic structure and function in MFS mice^9^. We also reported that doxycycline was more effective than atenolol (a common blood pressure lowering medication recommended in MFS patients) in preventing thoracic aortic aneurysm in mice^9^. However, the long-term *in vivo* effects of MMP inhibition on the progression of aneurysm, aortic function and wall stiffness by a sub-antibiotic dose of doxycycline, as well as its effects on elastic fiber ultrastructure s in the ECM of aortic wall have not yet been investigated. The present study was thus designed to estimate the long-term effects of a low dose doxycycline regimen on the biophysical properties of the aorta during the progression of aortic aneurysm *in vivo* using high-resolution imaging and high-frequency ultrasound system, and to additionally examine ultrastructural alterations in aortic elastic fiber using transmission electron microscopy (TEM). We hope that providing new knowledge about the potential use of long-term doxycycline treatment for delaying or blocking the progression of MFS-associated aortic aneurysms in the mouse model will underscore the rationale and warrant a similar clinical trial in human Marfan patients.

## Materials and Methods

The information written in this section was mainly excerpted and modified from the first author’s published graduate thesis, which was submitted to The Faculty of Graduate Studies at the University of British Columbia as part of requirements for the completion of the first author’s doctorate degree^10^.

### Experimental animals and treatments timeline

For the animal study, we used a transgenic mouse model, harboring an *Fbn1* allele encoding mutation C1041G (a cysteine substitution Cys^1041^→Gly), in an epidermal growth factor–like domain of fibrillin-1 (*Fbn1*^C1039G/+^)^6–8,11–13^. C57BL/6 wild-type mice were bred with heterozygous mice to generate control (Ctrl, *Fbn1*^+/+^) and Marfan (MFS, *Fbn1*^C1039G/+^) mice, which were housed in the institutional animal facility with standard animal room conditions (25 °C, 12-hour light-dark cycles, ≤5 animals in a cage). All animals used in this study were cared for in compliance with the Guide for the Care and Use of Laboratory Animals (www.nap.edu/catalog/5140.html); and all experimental approaches and procedures using live animals were performed according to an animal use protocol approved by the University of British Columbia institutional animal ethics board [reference number A15-0275].

Beginning at 6 weeks of age, thirteen control and twelve MFS mice were given a sub-antimicrobial dose of doxycycline hyclate (Alfa Aesar, Ward Hill, USA) in their drinking water (concentration = 0.24 g/L/day), which has been shown previously to be therapeutically active in inhibition MMPs activities^9^. Another group of twelve control and twelve MFS mice were given plain drinking water for comparison. Due to doxycycline sensitivity to light, prepared solutions were shielded from light; and were replenished and replaced every other day. During the study, treated and non-treated mice were subjected to ultrasound imaging (echocardiography) at 3, 6, 9, and 12 months of age in order to measure aortic diameters and pulse wave velocities (PWV).

### High-Resolution high-frequency ultrasound imaging

Information in this section was excerpted from the first author’s doctorate thesis^10^. “A high-resolution, high-contrast ultrasound imaging system Vevo® 2100 (VisualSonics, FUJIFILM, Toronto, Canada) equipped with a MS550 transducer was employed to conduct longitudinal experiments, to measure aortic diameters (indication of aortic dilatation), left ventricular (LV) mass (indication of LV hypertrophy), and PWV (indication of aortic elasticity/stiffness) in mice as described previously^14^. The central frequency of the transducer used for imaging was 40 MHz. The transducer also had a focal length of 7.0 mm with a frame rate of 557 fps (single zone, 5.08 mm width, B-mode). Two-dimensional (2D) images were recorded using the maximum field of view of 14.1 × 15.0 mm with a spatial resolution of 90 μm (lateral) by 40 μm (axial)”^10,14^.

“The aortic diameters, LV mass, and PWV were measured by ultrasound imaging system as previously described in detail^14^. Briefly, the experimental animal was anesthetized in an induction chamber using 3% isoflurane (Baxter Corporation, Mississauga, Canada) and 1 L/min 100% oxygen for 1–2 minutes. After testing the anesthesia state by confirming the loss of its righting reflex, the animal was laid supine on a heated platform with its nose enveloped in a nose cone to maintain anesthetized by 1.5–2% isoflurane^15,16^. Heart rate and respiratory rate were being monitored during the echocardiography procedure, as well as the electrocardiogram (ECG), which was measured by connecting the limbs of the mouse with ECG electrodes that were imbedded inside the platform. In addition, body temperature was also monitored through a rectal probe, which was maintained at 36–38 °C with a heating lamp”^10^.

Aortic diameters at three different regions of the aortic root (i.e. aortic annulus as L1, sinuses of Valsalva as L2, and sinotubular junctions as L3) were measured from the B-mode aortic arch view through the progression of the aortic root dilatation in the same experimental mouse. The 2D echocardiography method used for LV mass estimation is the area-length and truncated ellipsoid method. “The ascending and descending aortic peak velocities were measured from the PW Doppler-mode aortic arch view. PWV was calculated indirectly from the parameters obtained from the B-mode and Doppler-mode aortic arch view, by the formula: PWV = aortic arch distance/transit time (cm.s^−1^)^17^. The aortic arch distance was measured as d–d_0_ (mm) between the 2 sample volume positions, ascending and descending aorta labeled as d_0_ and d, respectively, along the central axis of aortic arch on the B-mode image. The PW Doppler-mode sample volume was placed in the ascending aorta and the time from the onset of the QRS complex to the onset of the ascending aortic Doppler wave form was measured as T_1_. Meanwhile, when the PW Doppler-mode sample volume was placed as distal as possible in the descending aorta, the time from the onset of the QRS complex to the onset of the ascending aortic Doppler wave form was measured as T_2_. The means for T_1_ and T_2_ were calculated from 10 cardiac cycles, and the transit time was calculated by T_2_ − T_1_ (ms). Therefore, indirect measurement of PWV was calculated by the equation of PWV = [d − d_0_]/[T_2_ − T_1_]”^10^.

### Aorta tissues preparation for histological staining

At 12 months of age, mice (n = 4) from different experimental groups were anaesthetized by inhalation of 3% of isoflurane, and the adequacy of anesthesia was confirmed by pedal reflex. Mice were then sacrificed by cervical dislocation. The ascending aorta (~10–14 mm) was dissected from treated and non-treated control and MFS mice, fixed in 10% buffered formalin for 48 hours, immersed in 70% ethanol overnight at 4 °C, and embedded in paraffin. Tissue specimens were transversely cut into 5 μm thick cross-sections and de paraffinized in xylene and rehydrated in graded ethanol. Elastic fibers of aortas were stained using Accustain® Elastic Stain kit (Sigma-Aldrich, St. Louis, USA) as described previously^10^. “Briefly, rehydrated tissue sections were placed in elastic stain solution for 10 minutes. Sections were rinsed in deionized water and then differentiated in working ferric chloride solution for 30–60 seconds. Differentiation was stopped with several changes of tap water. Sections were then rinsed in 95% alcohol to remove iodine and then in van Gieson solution for 3–5 minutes^18^. Following the staining procedure, samples were dehydrated through graded ethanol and xylene, and mounted with mounting medium with coverslip for light microscope imaging”^10^.

For immunohistological studies of the aortic wall, 5 µm aortic section from the ascending aorta were rehydrated and then stained with rabbit polyclonal primary antibody detecting total (pro + activated forms) of MMP-2 (ab37150, 1:100 dilution; Abcam, MA, USA), and rabbit polyclonal primary antibody detecting total (pro + activated forms) of MMP-9 (ab38898, 1:150 dilution; Abcam, MA, USA) using the EXPOSE Mouse Specific HRP/DAB Detection IHC kit (ab80436; Abcam, MA, USA), and as described in our previous published report^19^. Following multiple washes using Tris-buffered Saline (TBS), aortic sections were counter stained with Mayer’s hematoxylin (Sigma-Aldrich, MO, USA), and then imaged at 400X with the Olympus Vanox AH-3 microscope using AxioVision v.4.8.2. Imaging software. Images were analyzed using NIH Image-J 1.43j software that allowed for measuring the mean intensity of positive signal in the regions of interest (ROIs = 4 per section) after the subtraction of the background noise.

### Aorta tissues preparation for TEM imaging

Methods described in this section was excerpted from the first author’s doctorate thesis published by the University of British Columbia^10^.

“Thoracic aorta from 12-month old control and MFS mice (non-treated or treated with doxycycline, n = 4) were dissected and cleaned from fat and connective tissue. Specimens were washed in zero Ca^2+^ HEPES buffer, and the ascending aorta was transected above the level of the aortic valve, and transversely cut into three or four ring segments (length, 0.5 mm/each). The segments were then transferred into the glutaraldehyde fixative solution (contains 1 ml of 25% glutaraldehyde, 4 ml of ddH_2_O + 5 ml of 0.2 M sodium cacodylate buffer, pH = 7.4) and fixed for 1–1.5 hours. After the primary fixation, the ring segments were washed by 0.1 M sodium cacodylate buffer to remove the excessive glutaraldehyde, and then incubated in osmium tetroxide fixative solution (mixes with 5 ml of 0.2 M sodium cacodylate buffer, 2.5 ml of 6% of KFe_3_(CN)_6_, and 2.5 ml of 4% osmium) in room temperature for 2 hours. Osmium mix solution is used as a secondary fixative after glutaraldehyde fixative because its rate of penetration is too slow to prevent artifacts if used initially. After osmium binding the components of the aortic tissues, the ring segments became rigid and stable with the color turning brown. Following the fixation procedure, aortic segments were dehydrated through a series of graded acetone, and then infiltrated with epoxy resin and embedded in blocks for further processed for TEM”^10^.

### Quantitative TEM observations

Methods described in this section was excerpted from the first author’s doctorate thesis published by the University of British Columbia^10^.

“Twelve blocks of aorta specimens from three groups of experimental animals were randomly picked, and sectioned at 60–90 nm thickness on a Reichert ultra-microtome. The thin sections were stained, and then viewed on a Hitachi H7600 TEM. Digital TEM images were captured (15,000x) for a variety of extracellular matrix components, including elastic fibers, collagen, smooth muscle cells (SMCs), as well as the basal lamina apertures. For each cross-sectioned aorta observed, TEM images were acquired at four directions, north, south, east and west, and the quantitative analysis was performed. The gaps between the elastic fibers in a horizontal direction were spotted as the breakages, while the circumference or length (T) of each fragmented elastic fiber was traced along the fuzzy border up to the ends at which the thickness (D) was measured. The irregularity index (IR index) was introduced for morphometric calculation, by dividing the length of the borderlines by the width of each fragmented elastic fiber (IR index = T/D), and the average elastic fibers IRI was compared among the experimental animal groups. The higher the IR index, the more irregular and fragmented the elastic fibers will be”^10^.

### Statistical analysis

For the echocardiographic analysis, all measurements including aortic diameters and PWV were calculated and collected using the FUJIFILM VisualSonics® Vevo LAB software (FUJIFILM, Toronto, Canada), and averaged over five cardiac cycles to eliminate possible bias. A single investigator who was blinded to animal genotypes in experimental groups performed all image acquisitions and analyses. Experimental values were presented as means ± SEM, unless otherwise indicated. We used one-way analysis of variance (one-way ANOVA) to compare parameters among treatment groups followed by Tukey’s multiple-comparison tests. In order to compare values within the same experimental group but at different time points, we used one-way repeated measures ANOVA. When comparing values between two experimental groups, we used two-tailed Student’s *t*-test, with a *p* value of *p* < 0.05 considered to be statistically significant.

## Results

Information and data presented in this section was excerpted from the first author’s doctorate thesis^10^.

### Effects of long-term doxycycline intervention on pulse wave velocity and aortic wall stiffness in control and MFS mice

**Figure 1:**
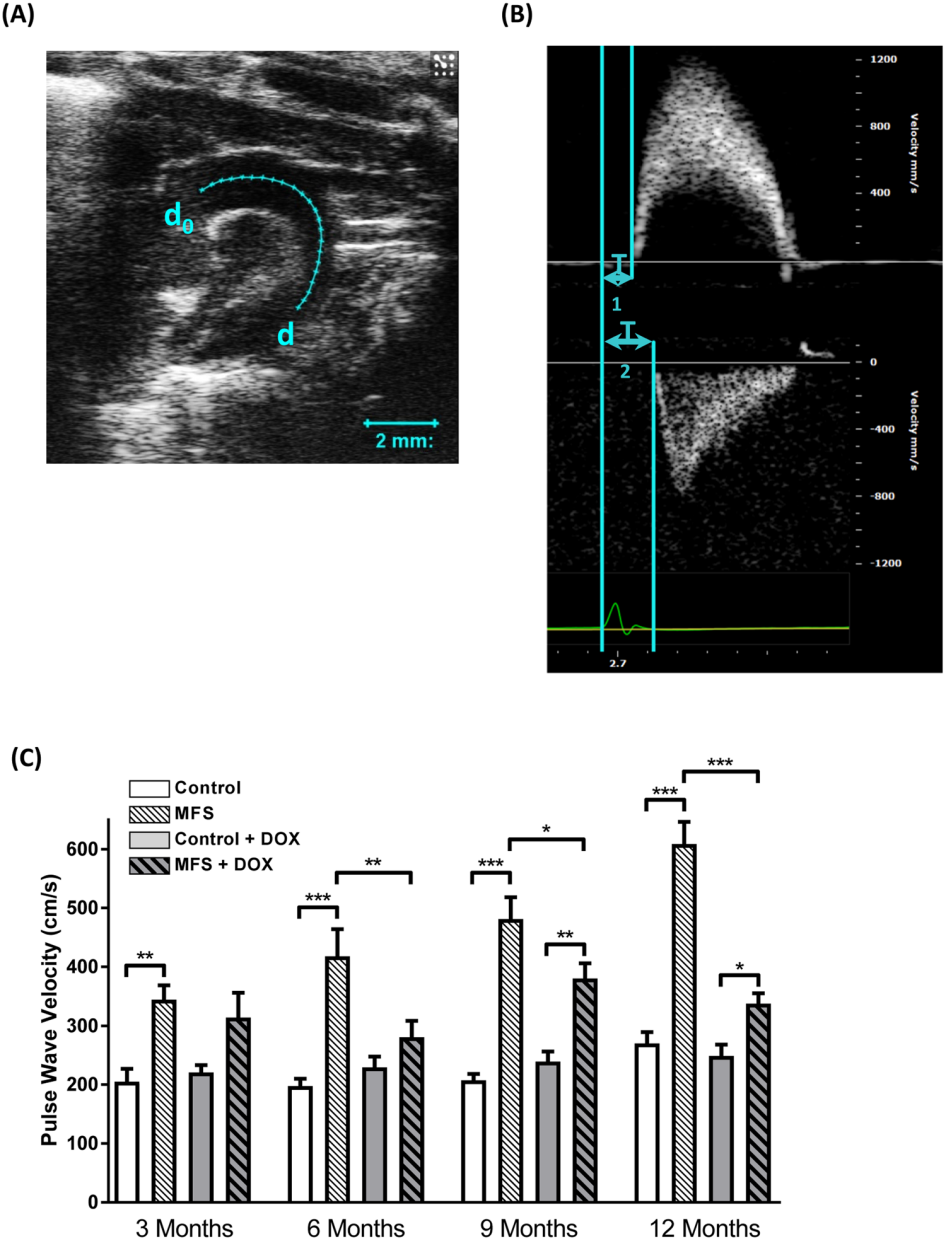
Effects of long-term doxycycline intervention on pulse wave velocity and aortic wall stiffness in control and MFS mice. (**A**) Control mouse: Aortic arch length from “d_0_” to “d” was measured on B-mode view as the distance; (**B**) Tracing recordings on pulse wave Doppler-mode view: time interval T_1_ in the ascending aorta (upper panel) and T_2_ descending aorta (lower panel). Pulse Wave Velocity formula: PWV = [d − d_0_]/[T_2_ − T_1_]; (**C**) PWV is significantly increased in MFS mice compared to controls without treatment at 3-month old (**p < 0.01); the difference in PWV is not significant in doxycycline-treated MFS mice compared to controls. PWV is significantly increased in non-treated MFS mice compared to controls at 6-month old (***p < 0.001), and doxycycline-treated MFS mice have significantly lower PWV values than non-treated MFS mice (**p < 0.01), But they do not display significant difference in PWV from either the treated or non-treated control mice, suggesting that long term doxycycline treatment exerted protective effects of on reduction of aortic stiffness in MFS mice compared to non-treated MFS mice starting from 6-month of age. PWV is significantly increased in MFS mice compared to controls without treatment at 9-month old (***p < 0.001); the doxycycline-treated MFS mice have significantly lower PWV than non-treated MFS mice (*p < 0.05), however, they show a significantly increased PWV compared to the treated control mice (**p < 0.01). The same findings as at 9-month old, but PWV in 12-month treated MFS mice continued to decrease further towards the levels in both treated and non-treated control mice, suggesting that long term doxycycline treatment extended protective effects of on reduction of aortic stiffness in MFS mice.

The non-invasive ultrasound imaging allows for a longitudinal study of the same animal subject through the progression of the aneurysm. Our measurements of PWV in mice (Fig. 1A,B) showed that in all age groups (3, 6, 9, and 12 months) PWV were higher in MFS mice as compared to age-match control subjects (Fig. 1C). Treatment with doxycycline had no significant effect on PWV in 3-month old MFS mice; however, the beneficial effects of doxycycline treatment in correcting the PWV in MFS mice was detectable starting at 6 months of age and continued until the mice reached the age of 12 months, underscoring the value of a long-term doxycycline regimen in correcting aortic function in MFS mice. Interestingly, doxycycline treatment had barely any effects on PWV measurements in control mice aorta, confirming that doxycycline is selectively targeting a pathologic pathway within the aortic wall of MFS mice without having any detectable effects on healthy aorta. Taken together, we conclude that in non-treated MFS mice, PWV increases significantly with age, indicating a gradual increase in aortic wall stiffness in MFS mice, a trend that is clearly reversed with doxycycline treatment (Supplementary Fig. S1A). It is noteworthy that although doxycycline treatment seems to significantly attenuate the age-related progression of aortic pathology in MFS mice, it is not able to completely block the progression of aortic stiffness in older (9 & 12 months old) MFS mice (Fig. 1C). Furthermore, a delayed increase in PWV was also observed in 12-month old control mice that was not significantly affected by doxycycline treatment, along with a slight increasing trend in PWV with age in treated control but not in treated MFS mice (Supplementary Fig. S1B).

### Effects of long-term doxycycline intervention on regional aortic root diameters in control and MFS mice

**Figure 2:**
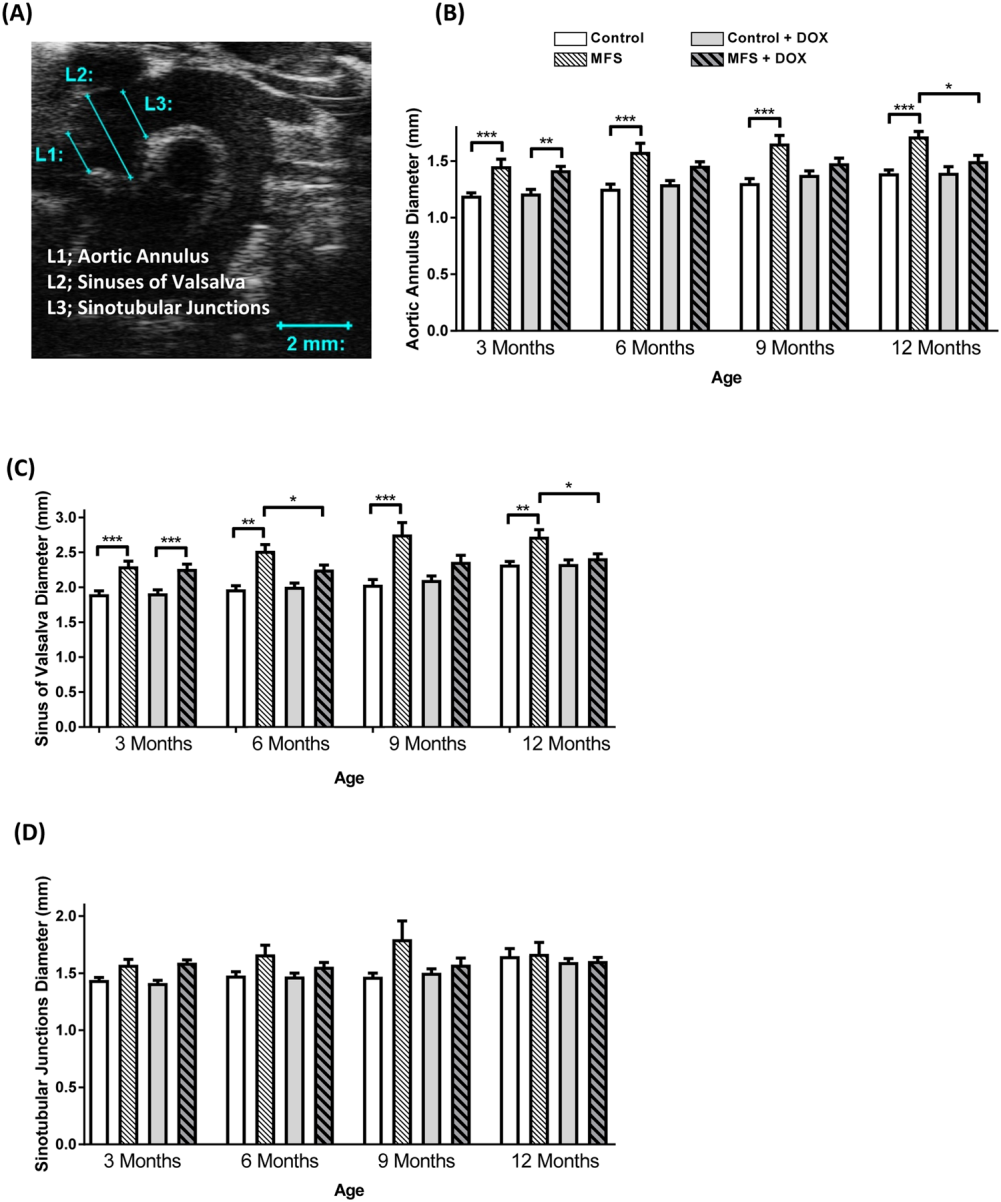
Effects of long-term doxycycline intervention on regional aortic root diameters in control and MFS mice. (**A**) Representative image of the B-mode view of a mouse aortic arch showing the diameters of three areas of interest, aortic annulus (L1), sinuses of Valsalva (L2), and sinotubular junctions (L3); (**B**) Aortic annulus diameters are significantly increased in MFS mice compared to controls with and without doxycycline treatment at 3-month old (**p < 0.01). Aortic diameters of the treated Marfan mice are not different from those in controls at age of 6-, 9-month old, while the aortas are all significantly wider in MFS mice without doxycycline treatment (***p < 0.001), suggesting severe aortic root dilation. Diameters of the treated Marfan mice are significantly decreased compared to non-treated MFS mice at age of 12-month old (*p < 0.05). The diameters also show no difference than treated control mice. (**C**) Aortic diameters at sinuses of Valsalva of the treated Marfan mice are not different from those in controls at age of 6-, 9-month old, while the aortas are all significantly wider in MFS mice without doxycycline treatment (**p < 0.01, ***p < 0.001, respectively). Furthermore, at 6-month old, treated MFS mice display significantly decreased aortic diameters compared to their age-matched MFS counterparts without treatment (*p < 0.05), suggesting potential protective effects of doxycycline on reduction of aortic dilation. (**D**) Measurements of diameters in sinotubular junctions that is a relatively distant loci of aorta from the left ventricle show that there are no differences in the aortic diameters between MFS and control mice, regardless of doxycycline treatment or not at all age groups, even though trends of dilated aortas in MFS mice can be observed. Diameters of the treated Marfan mice are significantly decreased compared to non-treated MFS mice at age of 12-month old (*p < 0.05). The diameters also show no difference than treated control mice (n = 12–13; Mean ± SEM; *p < 0.05, **p < 0.01, ***p < 0.001).

With the utilization of high-resolution ultrasound imaging, we used the B-mode view of aortic arch to measure aortic root diameters at three regions of interest (L1 = aortic annulus, L2 = sinuses of Valsalva and L3 = sinotubular junctions), in order to monitor the gradual progression of the aortic root aneurysm in the mouse model (Fig. 2A). At 3 months of age, values for root diameters at the aortic annulus were markedly increased in MFS groups as compared to controls with and without doxycycline intervention (*p* < 0.01, *p* < 0.001, respectively) (Fig. 2B). Aortic diameters of the treated MFS mice were not different from those in treated controls at age of 6- and 9-month old, although aortic diameters of non-treated MFS mice were significantly greater than their non-treated control counterparts (*p* < 0.001), confirming gradual and significant increases in aortic root diameters associated with aging in non-treated MFS mice. Following a ten-month intervention with doxycycline, and at 12 months of age, doxycycline suppressed the aortic dilatation at the aortic annulus in MFS mice (*p* < 0.05). Similarly, at 3, 6, 9 and 12 months, the diameters of the sinuses of Valsalva were shown to be markedly increased with aging in MFS mice as compared to control subjects (*p* < 0.001, <0.01, <0.001, <0.01, respectively) (Fig. 2C). Long-term doxycycline treatment reduced aortic diameters in 12-month old MFS mice to levels similar to the ones observed in non-treated 12-month control counterparts (*p* < 0.05). Moving further from the left ventricle, measurements of diameters at the sinotubular junction showed no marked differences between control and MFS mice (Fig. 2D), which were not affected by doxycycline treatment.

### Effects of long-term doxycycline intervention on MMP-2 and MMP-9 expression in the aortic wall of control and MFS mice

**Figure 3:**
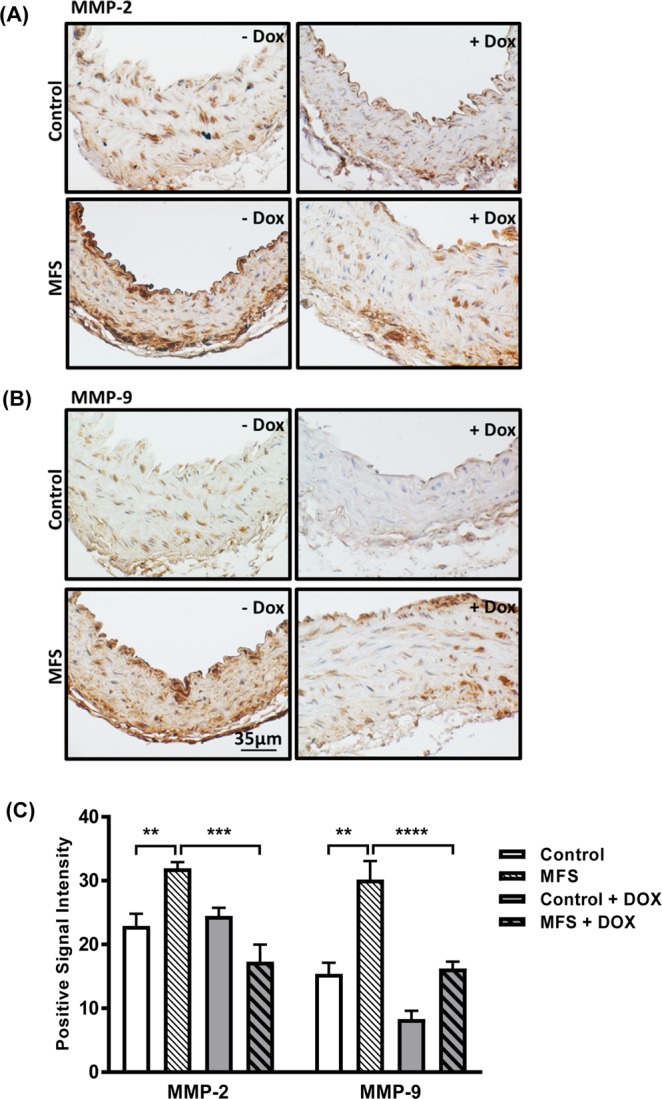
Effects of long-term doxycycline intervention on MMP-2 and MMP-9 expression in the aortic wall of control and MFS mice. Representative immunohistochemistry staining of total (**A**) MMP-2 and (**B**) MMP-9 expression levels within the cross sections of aortic wall isolated from 12-month old control and MFS mice in the presence and absence of doxycycline treatment. (**C**) Bar graphs representing quantification of MMP-2 and -9 expression levels within the aortic wall sections isolated from 12-month old control and MFS mice in the presence and absence of doxycycline treatment (n = 5; Mean ± SEM; *p < 0.05, **p < 0.01, ***p < 0.001).

We have shown that doxycycline treatment could slow down the progression of aortic root growth and reverses the observed increase in PWV in MFS mice aorta. To confirm that the observed protective effects were mediated through effects on MMPs signaling, we measured the expression levels of MMP-2 and MMP-9 within the aortic sections isolated from 12-month old control and MFS mice treated with sham or doxycycline (Fig. 3A,B). Our data confirm previous reports by us and others^9,20–22^ that MMP-2 and MMP-9 expression are significantly higher in MFS mice aortic wall as compared to age-matched controls (*p* < 0.05). We have also shown that doxycycline is effective in decreasing expression levels for both MMP-2 and MMP-9 within the aortic wall of MFS mice (*p* < 0.01) (Fig. 3C). These observations further confirm that MMP-2 and MMP-9 are involved in the pathogenesis of aortic aneurysm in MFS, and that doxycycline prevents aneurysm progression mainly through its inhibitory downstream effects on MMP-2 and MMP-9 production in the aortic wall.

### Correlations between aortic structure and function over time in treated and non-treated control and MFS mice

**Figure 4:**
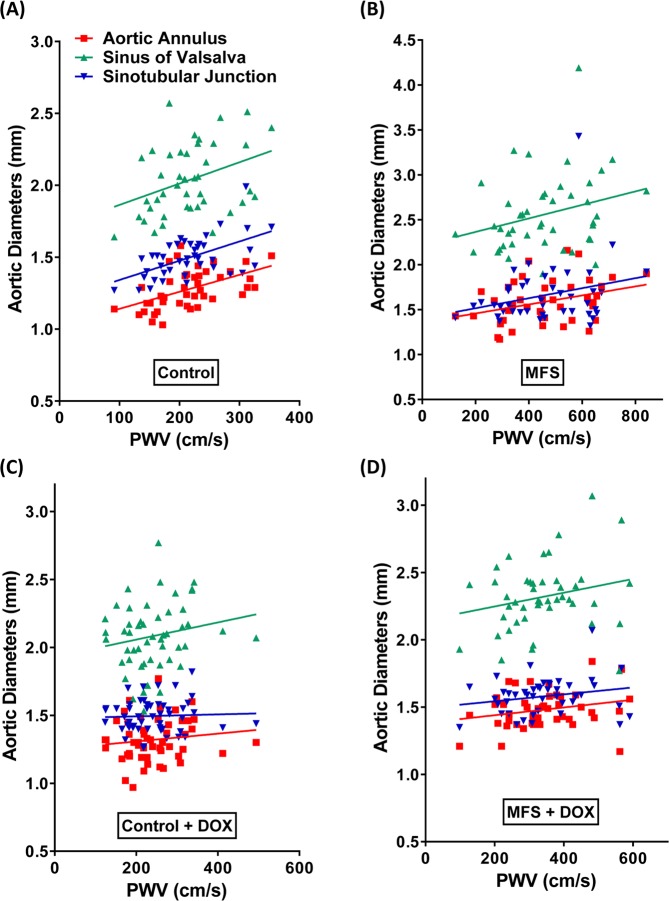
Correlations between aortic structure and function over time in treated and non-treated control and MFS mice. Linear regressions presenting the correlations between aortic diameters measurements and PWV in (**A**) non-treated control mice, (**B**) non-treated MFS mice, (**C**) doxycycline-treated control mice, and (**D**) doxycycline-treated MFS mice. The gap of the linear regressions between sinotubular junction and aortic root is narrowed, while the gap between these two regions and sinus of Valsalva is wider, in MFS mice compared to controls, regardless on doxycycline treatment or not. Out of three aortic regions, correlation in the region of sinus of Valsalva stands out as the highest level and warrants further investigation.

Our further analysis of possible correlations between aortic diameters in all three regions of aortic roots and PWV revealed positive linear regressions among the experimental groups regardless of the presence of absence of doxycycline treatment (Fig. 4). Interestingly, the gap between linear regressions for sinotubular junction and aortic annulus is narrowed in MFS mice (Y Intercept: 1.404 ± 0.1560 vs. 1.357 ± 0.1051, Fig. 4B) as compared to controls (Y Intercept: 1.208 ± 0.0693 vs. 1.023 ± 0.0687, Fig. 4A). However, it is evident that the gap is widening between these two regions and sinus of Valsalva in both control and MFS mice, respectively (Y Intercept: 1.715 ± 0.1338 and 2.214 ± 0.1932). A high correlation is evident in the region of sinus of Valsalva, indicating that this area is relatively more susceptible to the progressive structural and functional changes associated with the progression of aneurysm (R^2^ = 0.78, Fig. 4B), and is more likely responsive to a long-term doxycycline treatment (Fig. 4D). The observed correlations with age and doxycycline intervention at the sinus of Valsalva warrant further investigation.

**Figure 5:**
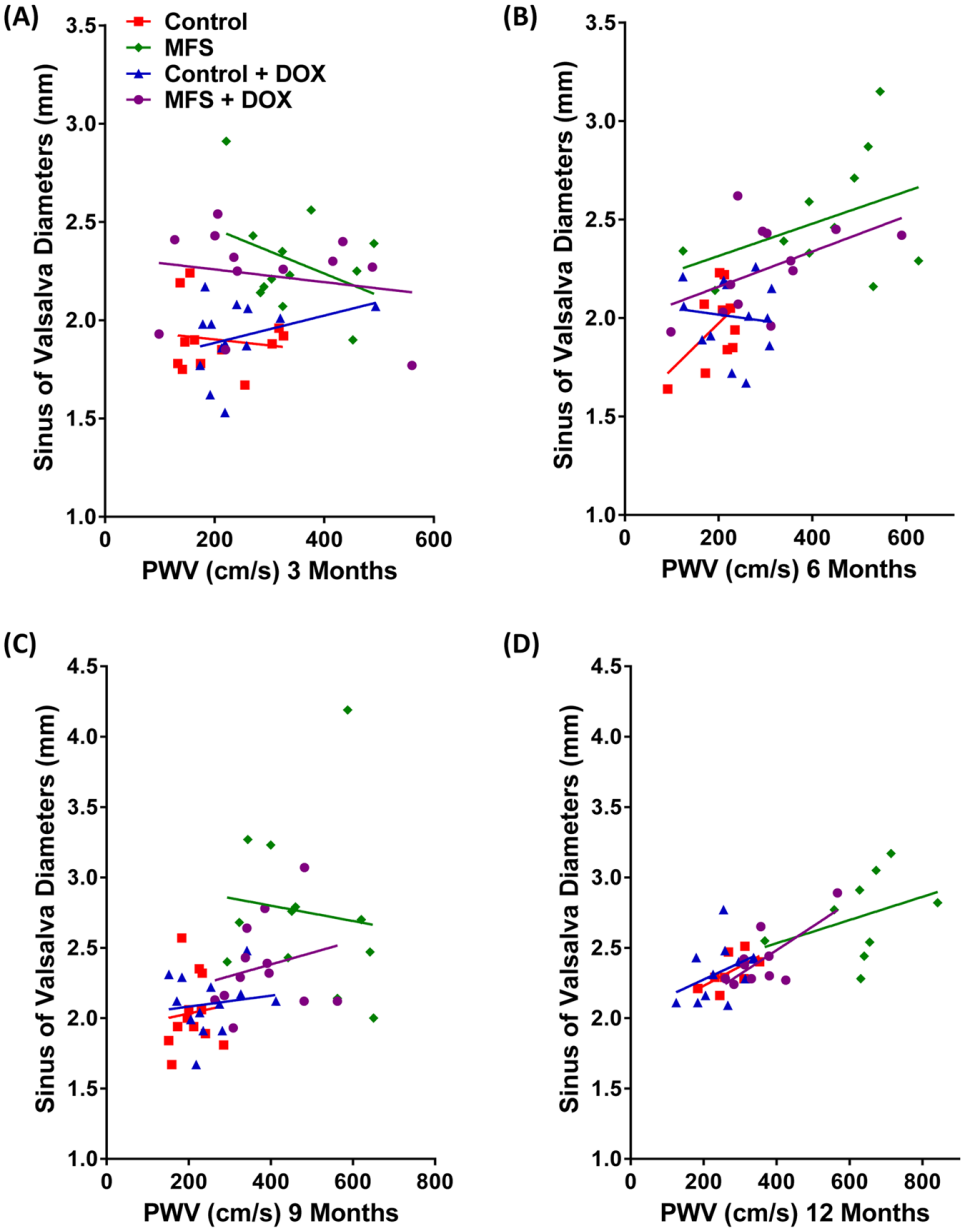
Correlations between PWV and aortic diameters at the region of sinus of Valsalva in different age groups. (**A**) At 3 months of age, there is no positive correlation with PWV in control and MFS mice in the presence or absence of doxycycline treatment. (**B**) At 6 months of age, there is a positive correlation with PWV in non-treated control mice (R^2^ = 0.96), as well as in non-treated and treated MFS mice. Linear regression level in treated MFS mice falls under the non-treated MFS mice starting at 6-month of age. (**C**) At 9-month old, correlation between PWV and aortic diameters continues to fall in treated MFS mice, while non-treated MFS remain dominant at the higher range. (**D**) At 12-month old, linear regression of the correlation in treated MFS group falls down to the level that is similar to the non-treated and treated control mice, while in non-MFS group it continues to stay at the higher level.

“With respect to measurements at the sinus of Valsalva, our data show that at 3 months of age, aortic diameters are not positively correlated with PWV in control and MFS mice regardless of doxycycline intervention (Fig. 5A). “However, at 6 months of age, a positive correlation is found between aortic diameters and PWV in non-treated control mice (R^2^ = 0.96, Fig. 5B), as well as in both treated and non-treated MFS mice. As we move from 6-month old to 9-month old group, linear regression level in treated MFS mice starts to fall below the non-treated MFS mice (Fig. 5C), while the latter ones remain dominant at the higher scale. Based on this observation, we can suggest that doxycycline may be effective in suppressing aortic dilatation after two months of treatment, while a wider and stiffer aorta is developed in non-treated MFS mice. Eventually, at the end of the experiment, when mice reach 12 months of age, linear regression level in treated MFS group falls down to the level of the control groups (Fig. 5D). However, the non-treated MFS group remains at the higher range, suggesting that long-term doxycycline treatment may improve both the aortic structure and function, particularly, at the sinus of Valsalva, mainly by blocking the aortic root growth and correcting the aortic wall elasticity”^10^.

### Comparison of cardiovascular gross structure and elastic morphology in treated and non-treated control and MFS mice

**Figure 6:**
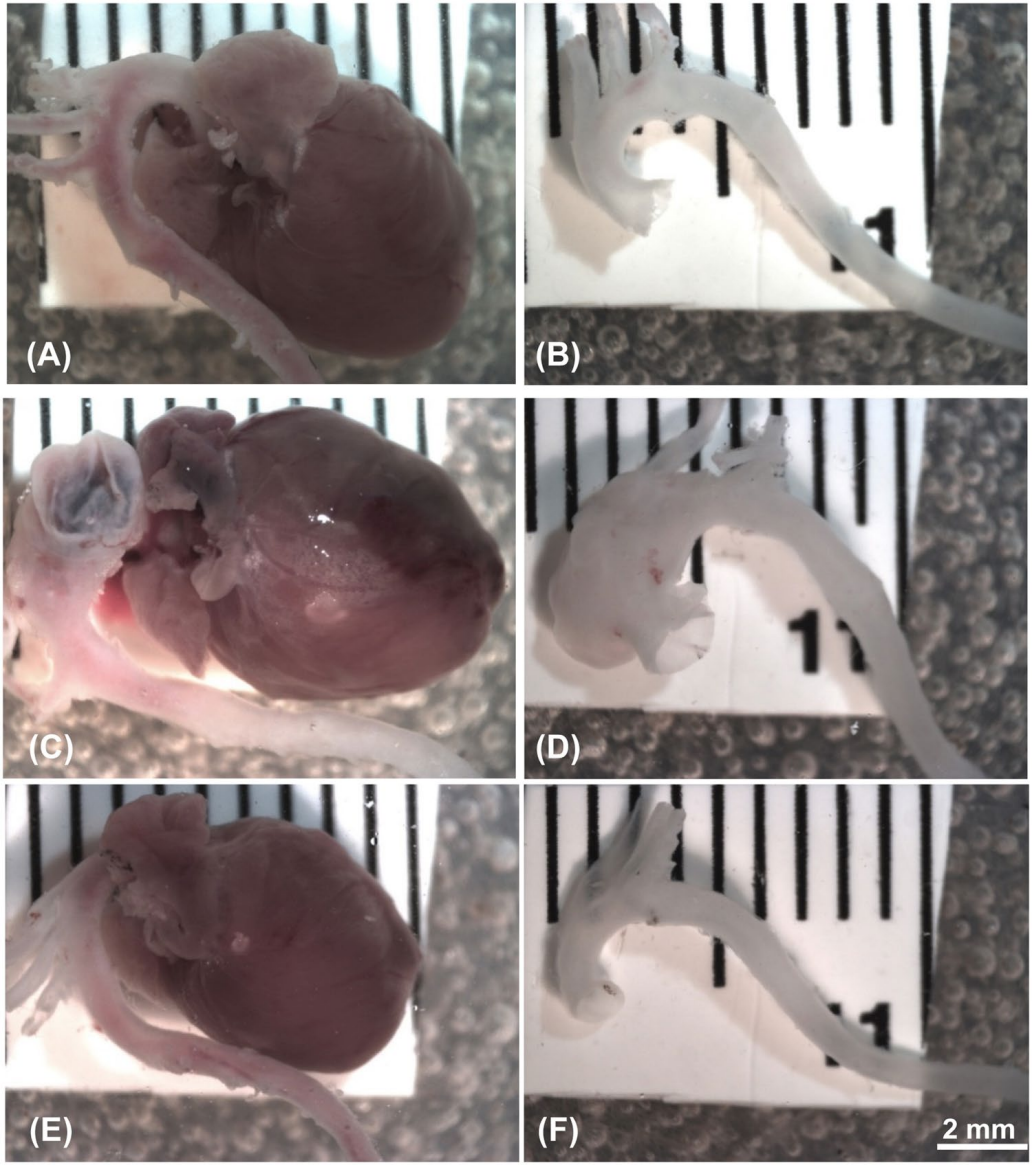
Comparison of cardiovascular gross structure and elastic morphology in treated and non-treated control and MFS mice. Representative images of dissected heart and aorta samples from (**A**,**B**) non-treated control mouse, (**C**,**D**) non-treated Marfan mouse, and (**E**,**F**) doxycycline-treated Marfan mouse at 12 months of age. (**A**,**B**) Intact mouse aorta with the typical three major branches in the aortic arch, which are, from proximal to distal, the brachiocephalic trunk, the left common carotid artery, and the left subclavian artery, attached to the normal shape of a control mouse heart. (**C**,**D**) Severe aortic dilation in MFS mouse without doxycycline treatment, a “balloon-like” dilation is observed prior to the aortic arch three branches. The left ventricle of MFS mouse heart becomes dilated. (**E**,**F**) Relatively normal shape and size aorta and heart can be observed in MFS mouse with doxycycline treatment.

**Figure 7:**
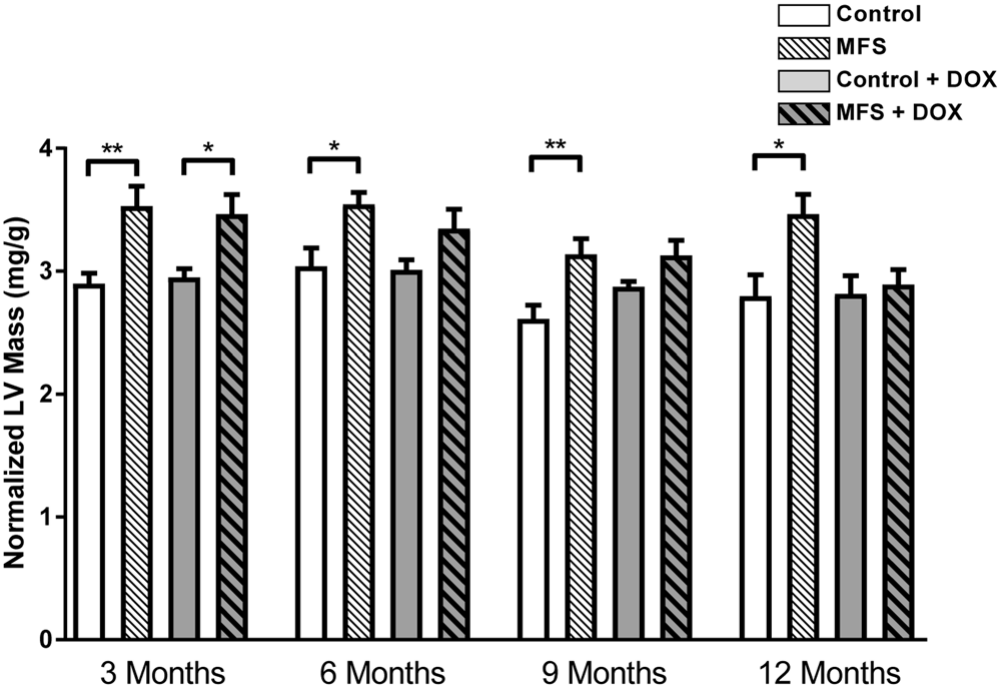
Measurements of normalized LV mass in treated and non-treated control and MFS mice. Without doxycycline treatment, Marfan mice showed significant increase in normalized LV mass compared to controls at 3, 6, 9, and 12 months of age (**p < 0.01, *p < 0.05, respectively). However, with doxycycline treatment, except for 3-month groups that LV mass was significantly increased in Marfan mice (**p < 0.01), no difference between treated Marfan and control mice from 6 months on, suggesting potential protective effects of doxycycline on reduction of LV mass (n = 12–13; Mean ± SEM).

Cardiovascular gross structures were compared in isolated samples from experimental animal groups at 12 months of age (Fig. 6). Normal intact aorta isolated from control mice show all three major branches normally observed in the aortic arch including, from proximal to distal, the brachiocephalic trunk, the left common carotid artery, and the left subclavian artery (Fig. 6A,B). In a non-treated MFS mouse, a balloon-like bulge, which is indicative of significant dilatation in the aortic root area, is evident with obvious signs of left ventricular hypertrophy in the isolated heart (Fig. 6C,D). With doxycycline intervention, the cardiovascular gross structure is normalized, with both aorta and heart displaying relatively normal shapes and sizes similar to those observed in the control mouse (Fig. 6E,F). Furthermore, the LV mass measurements confirm the above mentioned gross structural features. As shown in Fig. 7, the normalized cardiac weights in MFS mice are significantly larger than controls at all ages (3, 6, 9, and 12 months, *p* < 0.01, *p* < 0.05, respectively). At 3 months of age, treated MFS mice seem to have larger normalized LV mass as compared to age-matched control mic, however as mic age, no significant difference is detectable between treated MFS and control mice, suggesting possible protective effects for doxycycline on reduction of LV mass in MFS mice. This observation confirms our previously published report of increases in LV posterior and interior wall thickness and LV mass in MFS mice as compared to age- and sex-matched control subjects^14^.

**Figure 8:**
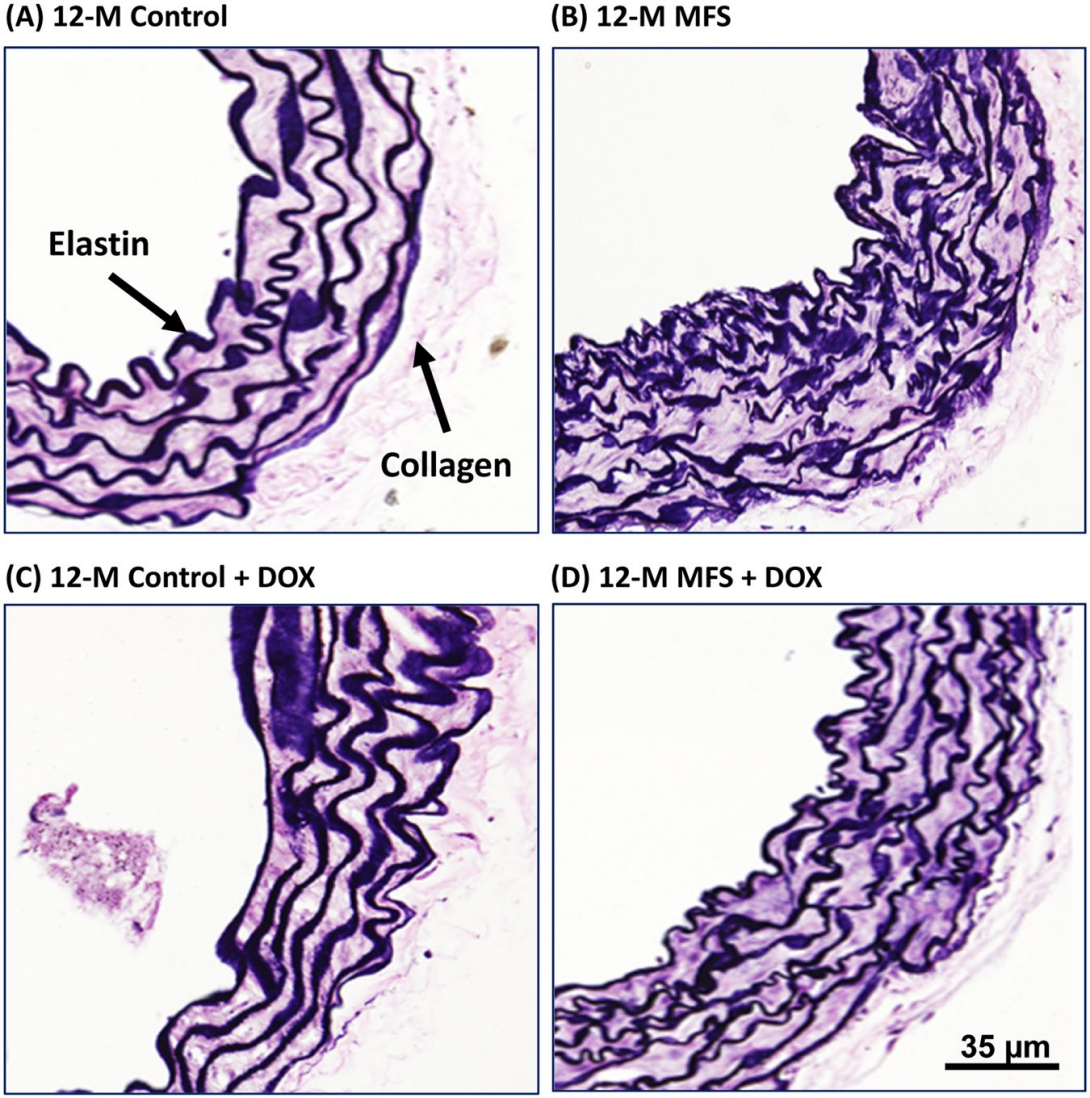
Representative images of van Gieson’s staining of the aortic root in treated and non-treated control and MFS mice. (**A**) Non-treated control, (**B**) non-treated Marfan, (**C**) doxycycline-treated control, and (**D**) doxycycline-treated Marfan mice at 12-month of age illustrate elastin (dark blue/purple) and collagen (light pink). Long-term doxycycline treatment shows protective effects on maintaining elastin fibers organization and aortic wall integrity on Marfan mice; however, it seems that this treatment cannot completely reverse the morphology as in Marfan mice.

In order to determine the morphology and structural changes in elastin fibers in the mouse ascending aorta, van Gieson’s staining was used in the aortic cross sections processed from the experimental mice at the age of 12 months old. As shown in Fig. 8, elastin fibers are stained in dark blue/purple, and collagen in light pink. The zigzag pattern of medial elastic lamella appears normal and preserved in control mice regardless of intervention (Fig. 8A,C), but elastin fibers within the medial layer of aortic sections isolated from non-treated MFS mice display severe fragmentation and disorganization (Fig. 8B). Long-term doxycycline treatment reduces elastin degradation and fragmentation in MFS mice aorta (Fig. 8D) as compared to non-treated groups, further confirming our previous published observations in MFS mice^5^.

### Ultrastructural changes at elastin break points in aortic section isolated from treated and non-treated control and MFS mice

**Figure 9:**
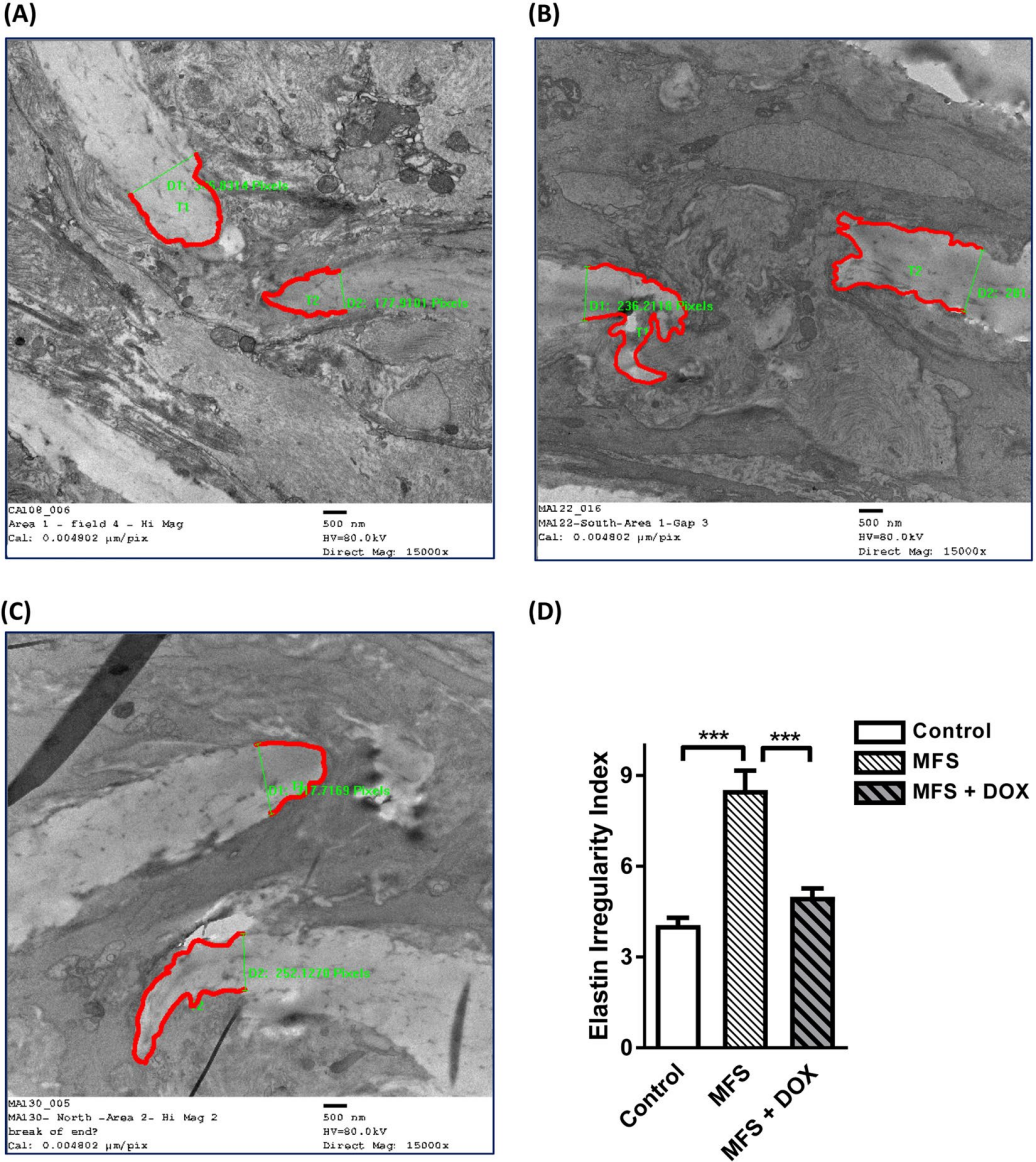
Ultrastructural changes at elastin break points in aortic section isolated from treated and non-treated control and MFS mice. Representative TEM images of ultrastructure of aortic elastin from (**A**) non-treated control mice, (**B**) non-treated Marfan mice, and (**C**) doxycycline-treated Marfan mice at 12 months of age. The boarder of the broken elastic fibers is relatively clean and sharp in control aorta; while irregular and fuzzy boarder can be observed in MFS mice. However, with long-term doxycycline treatment, it seems that the elastic fibers regain a normal shape on boarder lines, reversing the irregularities to the levels as those in their control counterparts. (**D**) Bar graphs showing the significance of observed differences in elastin irregularities index (circumference/width) in control, and non-treated and treated MFS mouse aorta. The irregularities index of elastin is significantly increased in MFS mice compared to controls (***p < 0.001), while elastic fibers in doxycycline-treated MFS mice aorta have significantly lower irregularities index than non-treated MFS mice (***p < 0.001), normalizing the irregularities to the levels observed in control mice (n = 4; Mean ± SEM).

“Our previous quantitative analysis of elastin structure in MFS aorta using multiphoton imaging demonstrated that elastic fiber length in all three aortic sections (ascending, arch, and descending) was significantly decreased in MFS mice compared to controls^13^. In addition, we reported that elastin orientation indices in MFS ascending aorta were lower^13^. Breakage and loss of elastic fiber integrity are believed to be the main underlying cause of aortic root aneurysm in patients with MFS. The irregular and shortened borderlines of elastic fibers could be the result of elevated levels of MMPs and their proteolytic activity. Therefore, TEM was used to further investigate whether the changes in the ultrastructure of the elastin fiber ends at the region of sinus of Valsalva of ascending aorta might be associated with the increase in breakage seen in MFS. Representative electron microscopy images of ultrastructural property of elastin fiber broken ends in aortic sections isolated from experimental subjects are presented in Fig. 9 and Supplementary Fig. S2. As shown in these images, the borders of the elastic fibers are relatively clean and sharp in the aortic section isolated from the control mice (Fig. 9A); while irregular and fuzzy borders are observed in the aortic section isolated from a non-treated MFS mice (Fig. 9B)”^10^. In the aortic sections isolated from doxycycline-treated MFS mice, the elastic fibers retained the normal shape on the borderlines in MFS mice aorta, therefore reversing the irregularities of the elastic fibers back to what was observed in healthy control subjects (Fig. 9C).

“Apart from the visual and qualitative ultrastructure observations, quantitative analysis was also performed to compare the irregularities of elastic fibers in aortic sections isolated from control and MFS mice in the presence or absence of doxycycline treatment. The average irregularity index of elastic fibers is significantly increased in MFS aortas compared to controls in the absence of doxycycline treatment (Fig. 9D) (p < 0.001); while the doxycycline-treated MFS aortas have significantly lower irregularities index as compared to non-treated MFS groups (*p* < 0.001)”^10^.

This suggests that MMP-mediated elastin fibers degradation in MFS mice aorta was significantly blocked in response to long-term doxycycline treatment. Taken together, our qualitative and quantitative observations suggest that long-term treatment with sub-antibiotic dose of doxycycline can block elastin degradation and fragmentation by the endopeptidase as observed at the ultrastructural level.

## Discussion

Some of the discussions included in this section were excerpted from the first author’s doctorate thesis^10^.

Aortic root aneurysm is the most life-threatening and lethal complication of MFS^23^. “Loss of aortic wall elasticity and increased stiffness due to significant fragmentation of elastic fibers, are considered crucial and detrimental factors contributing to the progression of aortic aneurysm^7^. An increase in aortic diameter with age in humans has been widely reported in various clinical studies, and a clear-cut increase in aortic surface area with age has been shown in autopsy studies^24^, although to a lesser degree, cross-sectional studies of aortic diameter by both ultrasound^25–29^ and angiography^30^ also show a definite increase with age. The blood pressure change associated with aging may also play a role in aortic dilatation, adding some controversy to the competing effects of age^25^. Progressive aortic dilatation with age has also been reported in patients with cystic aortic medial necrosis in longitudinal studies. In this case, the rate of dilatation has been shown to slow down in response to antihypertensive agents such as β-adrenergic blockers”^31,10^.

Previous studies have also evaluated the effects of β-blockers on aortic aneurysm development in MFS patients^31–38^. It is believed that the potential beneficial effects of β-blockers result from a reduction in dP/dt, which reduces aortic wall stress due to their negative inotropic and chronotropic effects. However, the precise mechanism underlying the protective effects of β-blockers has not yet been elucidated, and studies on their impact on aortic wall elastic properties have shown contradicting results^39–42^. Over the past decade, scientific findings about the roles that the renin–angiotensin system may play during the progression of aortic dilatation in MFS, and its contribution to the pathogenies of the disease, has been evolving. Angiotensin-II (Ang-II) type 1 receptor (AT1R) blockers were considered to be potential alternatives to the conventional and recommended treatment with β-blockers. In the aortic wall, Ang-II stimulates the proliferation of SMCs, induces fibrosis and blocks the programmed cell death or apoptosis, through binding to the AT1R. However, Ang-II association with the Ang-II type 2 receptor (AT2R) seems to have an anti-proliferative effect^43^. It is suggested that the effects of Ang-II are mainly mediated through transforming growth factor beta (TGF-β) signaling, leading to downstream increase of various signaling proteins such as MMPs that contribute to the destructive downstream events leading to aneurysm progression^43,44^. Previous studies in the mouse models showed that many phenotypic manifestations could be attributed to an increase in the activity of TGF-β^45–47^, and that treatments with antibodies against TGF-β reportedly prevented the development of myxomatous mitral valve disease and emphysematous abnormalities in mouse models of MFS^46,47^. Selective blockade of the AT1R with losartan also decreases TGF-β levels, thus inhibiting some of the mechanisms involved in the pathogenesis of MFS^45^. However, even though it has been shown that losartan can be effective in slowing down the progression of aortic aneurysm in the mouse model, the overall results of a meta-analysis of six clinical studies in human patients reported no benefits with respect to clinical outcomes^48^. On the other hand, two small randomized trials published previously showed beneficial effects of the general inhibitor of MMPs, doxycycline, in patients presented with progressive abdominal aortic aneurysms (AAAs)^49^, in which two weeks of preoperative treatment with doxycycline improved the proteolytic balance and decreased vascular inflammation in AAA samples^49^. However, despite the previous promising findings, doxycycline has not yet been formally tested in MFS patients presenting with aortic aneurysm; hence, further pre-clinical investigation is necessary to explicitly and carefully evaluate the *in vivo* effects of doxycycline on MFS-associated aortic complications.

Although MMP inhibition has been shown effective in preventing aneurysm formation in MFS mice, the studies reported previously were conducted *ex vivo* using organ chamber myography, and were not directly comparable with clinical data collected in human MFS patients using advanced *in vivo* imaging techniques. One specific example is the determination of vessel stiffness/elasticity by length-stress curves generated in a small vessel myograph. In this case, the applied stretch could cause irreversible damage to elastin and collagen construct within the aortic wall, a complication that is not encountered during *in vivo* echocardiography in human MFS patients. Hence, in this report, we sought to use non-invasive ultrasound imaging over time in the same experimental subjects with the hope of providing evidence that is more conclusive and provides a better rationale for putative clinical trials with doxycycline or other MMPs-inhibitors. The *in vivo* ultrasound imaging technique has the added benefit of simultaneous measurements of PWV as a reliable and clinically relevant indicator of aortic wall stiffness. In the present study, we established a developmental profile of gradual changes in the aortic root diameters in MFS mice at 3, 6, 9, and 12 months of age. It is noteworthy that drastically dilated aorta was observed in MFS mice at as early as 3 months of age, particularly, at the aortic annulus and sinus of Valsalva. This correlates with early detection of loss of elastic fiber organization in 3-month old MFS mouse aorta^13^. Treatment with doxycycline prevented the increase in aortic root diameters at the aortic annulus and sinus of Valsalva in the 6- and 12-month old treated MFS mice. Interestingly, when we looked at the possible correlations between PWV and aortic root diameters with the mouse age, we noticed that the correlation was only pronounced in the region of the sinus of Valsalva (Fig. 5), indicating that the aortic wall in the region of the sinus of Valsalva is relatively more susceptible and responsive to doxycycline treatment.

We and other research groups have shown that the progression of aortic aneurysm in the mouse model of MFS is associated with a significant increase in MMP-2 and -9 expression within the aortic wall^6–8^. MMPs are a family of zinc-containing, calcium-activated endopeptidases with proteolytic activity targeting the components of the ECM. They play important roles in controlling different physiological processes in the tissue, for instance vascular remodeling and angiogenesis, and can play critical roles in vascular diseases such as hypertension, atherosclerosis, aortic aneurysm, and varicose veins. MMP activity can be blocked by specific and non-specific inhibitors, including endogenous tissue inhibitors of MMPs (TIMPs), and pharmacological inhibitors, such as zinc chelators, marimastat and doxycycline. Doxycycline is known as a general and nonspecific inhibitor of MMPs even at sub-antimicrobial doses, and currently, is the only broadly available MMP inhibitor already in use in clinical practice^50,51^. Members of the MMP family, particularly MMP-2 and -9, and their inhibitors contribute to physiological turnover of ECM components, and re-organization and maturation of elastic and collagenous fibers^52–58^. Increased expression and activity of MMPs has been reported in human aortic aneurysm tissue^59–62^ and MFS mice aorta^5,6,8^. Some studies have also suggested that upregulation of MMP-2 in vascular smooth muscle cells plays an important role in MFS aneurysm development and progression^63,64^. In our study, we were able to show that doxycycline treatment could decrease MMP-2 and -9 expression within the aortic wall, while blocking aortic root growth and correcting aortic wall elasticity and PWV. These observations corroborate previous reports showing the effects of doxycycline on MMP expression levels in the mgR/mgR mouse model of MFS^20,65^.

A previous study that compared the effects of doxycycline and several non–antibiotic chemically modified tetracycline (CMTs) in a rat model of elastase-induced AAAs found that tetracycline derivatives could block the progression of AAAs in rats^66^. Another study in a rat model of connective tissue breakdown has also reported beneficial effects of tetracycline derivatives by inhibiting MMPs and other tissue-destructive pathways^67^. It has been reported that tetracycline can bind to Ca^2+^/calmodulin regulated phosphodiesterases responsible for cyclic AMP (cAMP) hydrolysis, and thereby affecting MMP expression and activity in some cells such as monocytes and cartilage cells^68,69^. A recent study has reported that doxycycline, at a nontoxic dose, can inhibit TGF-β1-induced MMP-9 production and activity in human corneal epithelial cells^70^.

“Aortic wall stiffness is a functional indicator of aortic structure that reflects the structural integrity of its major ECM components including collagenous and elastic fibers. Utilizing *ex vivo* approaches such as isometric small chamber myography, our laboratory previously reported an increase in aortic wall stiffness and lack of reversibility of elasticity in aortic rings isolated from 6- and 9-month old MFS mice. The reduced elasticity in MFS aorta could be attributed to elastin fiber fragmentation in aortic wall^5^. With the use of high-resolution echocardiographic imaging technique, the progressive aortic dilatation and stiffness can be studied non-invasively over time in both mouse models and patients with Marfan syndrome^14,40,71,72^. PWV that is believed to be a robust index of aortic wall stiffness can be measured non-invasively in human patients and the mouse model using high frequency ultrasound imaging, and has been demonstrated as the earliest predictor of cardiovascular risk in different populations, including elderly, hypertensive, diabetic and renal patients^73–76^. Bradley and colleagues^17^, who reported an increased PWV in pediatric MFS patients compared to normal subjects, were able to establish a reliable echocardiographic Doppler method, to indirectly measure the aortic wall stiffness in humans, that was later adapted by our group to reliably assess a variety of biophysical properties in the mouse model of MFS^14^. “Using these techniques, we have shown that beginning at 3 months of age, PWV is significantly increased in MFS mice compared to controls in all age groups without doxycycline intervention, with the increases ranging from 1.5-fold to over 2-fold at different ages. These observations are not only consistent with previous studies using echocardiography on MFS patients and mice^14,17,77–80^, but also expand the age groups and display an extended upward trend in PWV.” (Citation of JZC’s thesis) Most importantly, PWV is significantly higher in MFS aorta vs. controls beginning at 3 months of age, when the aortic root aneurysm is not yet detectable, suggesting that in MFS, loss of aortic wall elasticity precedes the aneurysm formation”^10^.

The correlation between aortic diameter and PWV at the sinus of Valsalva reveals a gradual deterioration of aortic wall structure that is associated with loss of function. Our data clearly confirm that in doxycycline-treated MFS mice, when compared to non-treated ones, the progression of aortic dilatation, particularly at the sinus of Valsalva, is delayed. We have also provided evidence that aortic aneurysm in MFS mice is associated with increase in LV mass, and that doxycycline seems to also have protective effects on cardiac structure by reducing LV mass in MFS mice.

Conventional van Gieson’s staining of the ascending aorta re-confirms our previous report showing significant loss of elastin fibers organization and their pronounced degradation and fragmentation in non-treated MFS mice when compared to controls^13^. We have previously reported that doxycycline intervention corrects elastin fiber organization, and preserves aortic wall integrity in MFS mice mainly through the inhibition of MMPs proteolytic activities, suggesting a causal relationship between enhanced MMP activity and elastin fiber fragmentation in MFS mice aorta^9^. Besides, previous reports have established that doxycycline can also inhibit experimental AAAs, and reduce MMP expression in the area of aneurysm^21,22,81^. In order to obtain more detailed information about the structural consequences of elastin degradation by MMP and its reversal by doxycycline, we performed electron microscopy on the affected regions of the aorta of MFS mice. TEM images show that the terminal ends of the elastic fibers are relatively sharp and clean in controls, but appear frayed and irregular in the MFS aorta. This conclusion was statistically validated by the introduction of the Irregularity Index. The difference appeared to be the result of enhanced elastolysis in the MFS aorta, since it was prevented by MMP inhibition with doxycycline. Summing up the above evidence, it is evident that doxycycline inhibition of MMPs provides beneficial effects to aortic structure and function in the region of sinus of Valsalva, which seems to be highly susceptible to aneurysm progression in MFS.

## Conclusion

In this longitudinal study, we have determined the beneficial effects of long-term intervention with sub-antibiotic dose of doxycycline on cardiac structure (LV mass) and aortic structure and function in a mouse model of MFS. We have concluded that doxycycline can significantly reduce aortic root dilatation, and correct PWV and aortic wall stiffness in MFS mice. In addition, this study provides evidence that doxycycline can significantly decrease the levels of MMP-2 and -9 expression and elastin fragmentation in the aortic wall of MFS mice, leading to a significant improvement in aortic wall elasticity (as measured by PWV). The ultrastructural changes of elastin in the ECM were also investigated and quantitatively analyzed by TEM imaging, and it was shown that the MFS-associated morphometric modifications of elastic fibers were drastically improved with doxycycline regimen.

### Limitation of the Study

The data presented herein may shed some light on the potential therapeutic value of doxycycline in the context of MFS-associated aortic aneurysm; hence, strengthen the rationale for designing similar clinical trials in MFS patients in the near future. However, it is critical to appreciate the concern for potential antibiotic resistance with the long-term use of doxycycline even at a very low and sub-antibiotic dose. While the concerns are theoretically valid, given the potential severity of the disease, it seems to be worthwhile to test the theory to find out if these potential problems occur. In addition, the same concern warrants further carefully designed experiments that would investigate the impact of long-term treatment with sub-antibiotic dose of doxycycline on the gut microbiome in the mouse model. This also brings forward the notion of using non-antibiotic MMP inhibitors that are now becoming available. It is of importance to emphasize that the presented study was designed with the aim of providing a solid proof-of-concept in an effort to establish the rationale for a clinical trial using MMP inhibitors in human MFS aneurysm patients. This is of much more importance and urgency, as the current pharmacologic treatments have not been very effective in eliminating the need for aortic replacement surgery, and most importantly in improving the quality of life for MFS patients.

## Supplementary information

Supplementary Figures

## Data Availability

The raw data and all microscopy images used to produce statistical data presented in this report will be available to interested readers, and can be obtained by contacting the corresponding author.
