# Supplementary material for: In vivo characterization of doxycycline-mediated protection of aortic function and structure in a mouse model of Marfan syndrome-associated aortic aneurysm
Source: Sci Rep. 2019 Feb 14;9:2071. doi: 10.1038/s41598-018-38235-6 (PMC6376062; doi:10.1038/s41598-018-38235-6)
Supplement: Supplementary file 1 — Supplementary Figures [file 41598_2018_38235_MOESM1_ESM.pdf]

Supplementary Information

In vivo characterization of doxycycline-mediated protection of aortic function and structure in a mouse model of Marfan syndrome-associated aortic aneurysm

Jason Z. Cui, Ling Lee, Xiaoye Sheng, Fanny Chu, Christine P. Gibson, Taline Aydinian, David C. Walker, George G. S. Sandor, Pascal Bernatchez, Glen F. Tibbits, Cornelis van Breemen, Mitra Esfandiarei

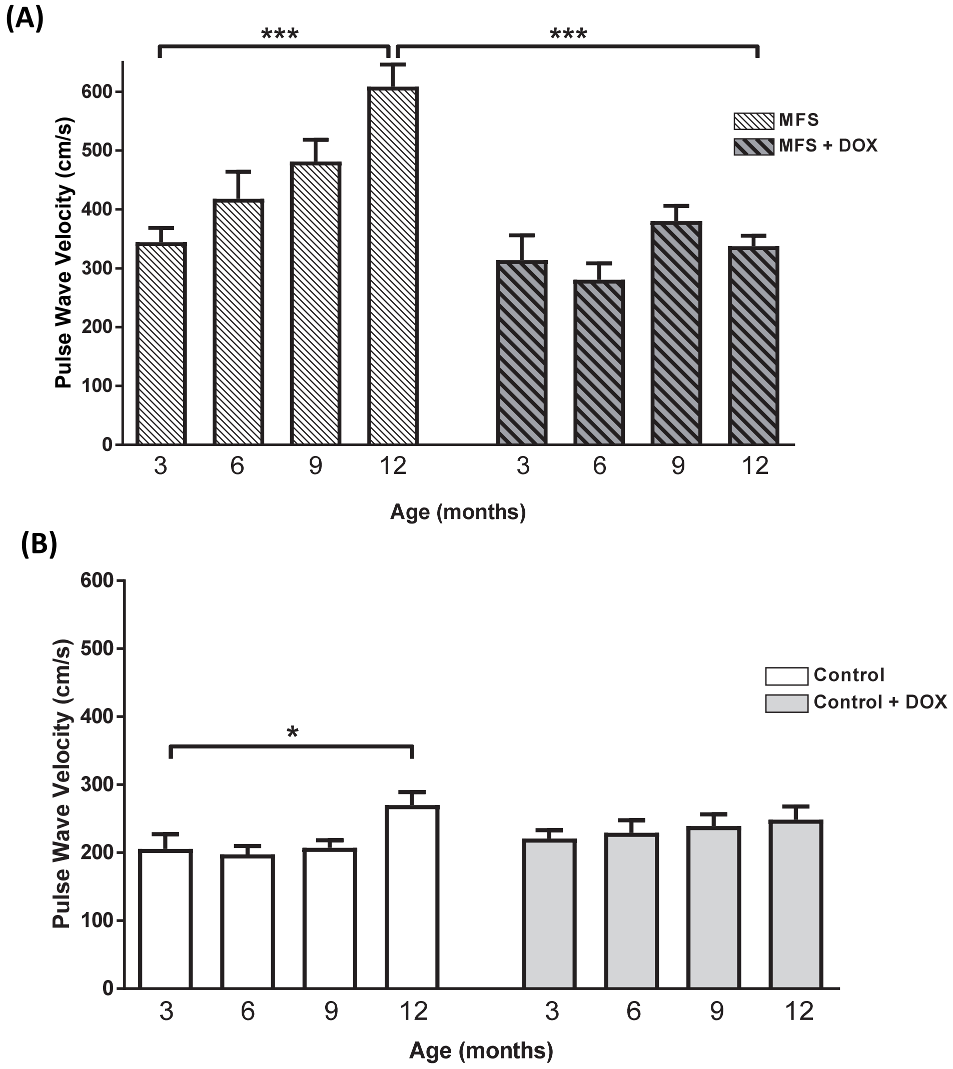

Figure 1S. Longitudinal measurements and comparison of pulse wave velocity in **(A)** Marfan and **(B)** Control mice in the absence and presence of doxycycline treatment based on the age groups. (n=12-13; Mean ± SEM; *p<0.05, **p<0.01, ***p<0.001).

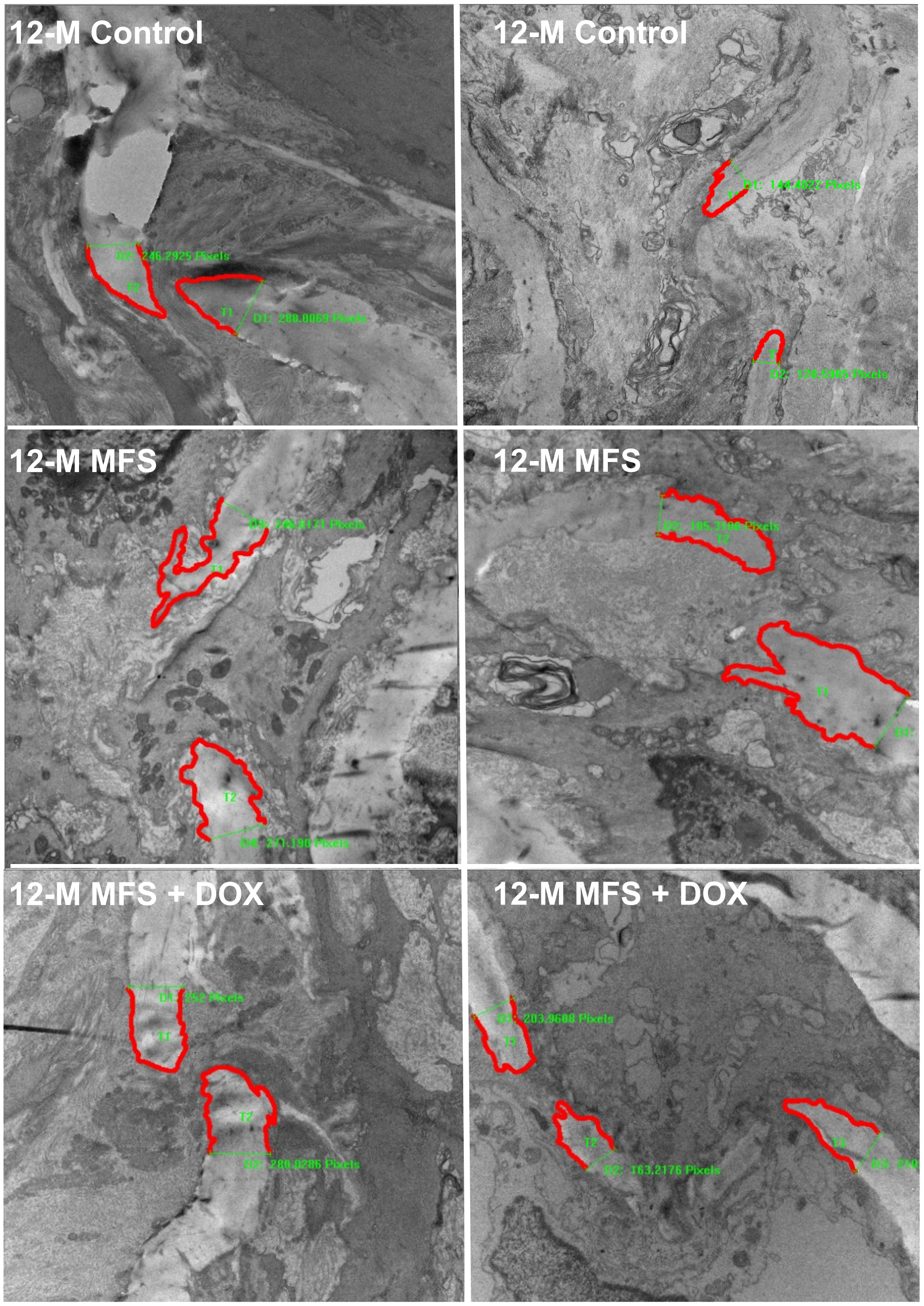

Figure S2. Supplementary representative TEM images (n=2) of ultrastructural changes at elastin break points in aortic section isolated from treated and non-treated control and MFS mice at 12 months of age.
